# Percutaneous Intervention in ST-Elevation Myocardial Infarction: Culprit-only or Complete Revascularization?

**DOI:** 10.5935/abc.20170174

**Published:** 2017-12

**Authors:** Ana Paula Susin Osório, Alexandre Schaan de Quadros, José Luiz da Costa Vieira, Vera Lucia Portal

**Affiliations:** Instituto de Cardiologia/Fundação Universitária de Cardiologia (IC/FUC), Porto Alegre, RS - Brazil

**Keywords:** Myocardial Infarction, Myocardial Revascularization, Percutaneous Coronary Intervention, Coronary Artery Disease, Stents

## Abstract

The best approach of multivessel coronary artery disease in the context of acute myocardial infarction with ST segment elevation and primary percutaneous coronary intervention is one of the main reasons for controversy in cardiology. Although the main global guidelines do not recommend routine complete revascularization in these patients, recent randomized clinical trials have demonstrated benefit of this approach in reducing cardiovascular outcomes. For this reason, an adequate review of this evidence is essential in order to establish scientifically based strategy and achieve better outcomes for these patients who present with acute myocardial infarction. This review aims to present objectively the most recent evidence available on this topic.

## Introduction

The primary percutaneous coronary intervention (PPCI) currently represents the treatment of choice for acute ST-Elevation Myocardial Infarction (STEMI).^1^ However, despite its undeniable benefit, some issues related to its appropriate use are still controversial.

Approximately 40 to 50% of patients with STEMI have multivascular coronary artery disease (CAD),^2^ although most of those individuals are asymptomatic until their acute manifestation.^3^ It is known that, when compared to patients with CAD with involvement of a single vase, they have higher rates of mortality and of recurrent non-fatal infarction incidence.^4,5^ Brazilian data, from the Cardiology Institute of Rio Grande do Sul, show that in a total of 2,469 patients treated due to STEMI during the period from 2010 to 2014, about 30 and 20% of them had two and three vessels affected, respectively. In a multivariate analysis, the three-vessel CAD was proven a strong predictor of mortality in 30 days (OR 3.39; 95%CI 1.47-3.87; p < 0.001).^6^

The long-term prognosis of acute myocardial infarction (AMI) associated with multivascular CAD is worse, probably due to a series of pathological mechanisms, such as: additional instability of other atherosclerotic plaques; impairment in myocardial perfusion caused by endothelial dysfunction; microvascular spasm or inflammation; and contractility reduction in non-infarcted areas. The worse log-term outcomes may also be related to the higher age of patients, in addition to more risk factors for atherosclerosis and decreased left ventricular function in individuals with multivascular CAD.^7^ In this context, the benefits of multiarterial revascularization may be related to decreased risk of new coronary occlusions, decreased total ischemic load and improves potential for collateral circulation.

Thus, there is an important questioning regarding the best PPCI strategy in these individuals: the treatment solely of the lesion responsible for the AMI or the complete revascularization, with angioplasty of stenosis in arteries not related to AMI.

Metanalyses of observational studies, mostly records, have demonstrated conflicting results when PPCI is performed in arteries which are not responsible for the AMI.^8-10^

Cavender et al.^8^ collected data from the National Cardiovascular Data Registry, an American registry of 708,481 hospital admissions during the years of 2004 to 2007, with the objective of determining the prevalence, the predictors and the in-hospital outcomes of complete revascularization in AMI. Patients submitted to the multivascular approach were more severe, with greater incidence of cardiogenic shock, heart failure (HF), left ventricular ejection fraction lower than 30% and impairment of the proximal anterior descending artery. In-hospital mortality was higher among those submitted to complete revascularization (7.9% *versus* 5.1%; p < 0.01). Patients in cardiogenic shock who received PCI of arteries not responsible for the AMI also had higher mortality (36.5% *versus* 27.8%; OR 1.54; 95%CI 1.22-1.95).

Bangalore et al.^9^ collected data from 19 studies (61,764 patients) evaluating multivascular AMI and CAD, with the objective to compare early (< 30 days) and late outcomes submitted to PCI solely of the responsible artery or complete revascularization. Of the 19 studies, only 2 were randomized. Patients who underwent the staged strategy were excluded. There was no significant difference in early mortality outcomes, AMI, cerebrovascular accident (CVA) and revascularization of the target vessel. In the long-term (2 ± 1.1 years), there was no difference in mortality, AMI, CVA, revascularization of the target vessel and stent thromboses, though there were reductions of 33% in mortality, 43% in the need of percutaneous intervention and 53% in the myocardial revascularization surgery. There was a significant reduction of adverse cardiovascular events when the complete revascularization strategy was used, when compared to the approach solely of the responsible artery (OR 0.60; 95%CI 0.50-0.72).

In metanalysis of 11 studies, Sethi et al.^10^ compared the outcomes of 4,640 patients submitted to complete revascularization during PPCI procedure or in the same hospitalization with 27,394 patients treated only with PPCI of the responsible artery by AMI. Only 2 of them were randomized clinical trials (RCTs), 8 were observational studies and 1 was a control case. Most patients were hemodynamically stable. There was no difference in relation to greater cardiovascular events (OR 0.95; 95%CI 0.47-1.90) and to long-term mortality (OR 1.10; 95%CI 0.76-1.59). There was heterogeneity among the studies, in addition to the absence of specific designs to answer this question.

Two great metanalyses of observational studies,^11,12^ with over 40,000 patients each, reported the complete approach during the PPCI procedure was associated with higher mortality, while the staged intervention (performed later during hospitalization or 30 after the acute event) was associated to reduced mortality. Small RCTs did not show improvement in either outcomes nor prognostic of patients treated with complete revascularization in acute context.^13,14^

In pair and network metanalysis, involving 4 prospective studies and 14 retrospective studies with a total of 40,280 patients, Vlaar et al.^11^ evaluated three revascularization strategies: 1) PCI solely of the artery responsible for the AMI; 2) immediate complete revascularization of one or more arteries not responsible for the AMI; 3) staged revascularization during hospitalization of one or more arteries not related to the AMI. In paired analysis, it could be observed that the staged revascularization associated to lower short- and long-term mortality, when compared to PCI of the responsible artery or immediate complete revascularization. Immediate complete revascularization had higher mortality rates in the short and long terms. Network metanalysis showed staged revascularization would consistently associate with lower mortality.

Similarly, Bainey et al.^12^ performed systematic reviews and metanalysis of 26 studies (46,324 patients) comparing revascularization strategies for multivascular CAD in AMI. Only 3 studies were randomized. There was no difference for in-hospital mortality when compared to PCI solely of the responsible artery with complete revascularization, however, this mortality was increased when the approach of other arteries was performed during the same procedure as the PPCI. Reduced in-hospital mortality was observed with complete staged revascularization. With the strategy of complete revascularization, there was a reduction in the long-term mortality and in the need for new interventions.

### Guidelines recommendations

The main guidelines on the treatment of STEMI have discouraged PCI of arteries not responsible for AMI. According to ESC Guidelines for the Management of Acute Myocardial Infarction in Patients Presenting with ST-Segment Elevation,^15^ published in 2012 by the European Society of Cardiology, there is no evidence for emergency intervention in lesions which are not responsible for AMI. The approach of multivascular CAD during PPCI is only justified in cases of cardiogenic shock with presence of multiple critical stenosis or of highly unstable lesions (angiographic signs of thrombi or rupture of the lesion) or of evidence of persistent ischemia in spite the angioplasty of the affected artery.

On the other hand, the American directive, published in 2013 (2013 ACCF/AHA Guideline for the Management of ST-Elevation Myocardial Infarction),^16^ considered as class III (causing damage) the angioplasty of arteries not responsible for AMI, in the context of PPCI in patients without hemodynamic compromise. However, with the newly observed evidences, especially after the publication of the PRAMI study,^17^ in 2013, an update of this guideline was published, considering class IIb the angioplasty of the artery which was not responsible for the PPCI or as staged procedure prior to hospital discharge.^18^

More recently, the Brazilian guideline directive (*V Diretriz da Sociedade Brasileira de Cardiologia sobre Tratamento do Infarto Agudo do Miocárdio com Supradesnível do Segmento ST*), published in 2015, recommends the percutaneous approach should be dedicated to the artery responsible for the AMI. The immediate revascularization of other arteries not responsible for the event during PPCI may be considered in patients selected, as in cases of less complicated severe stenosis located in the same coronary system related to the infected vessel and according to the criterious evaluation of the clinical and hemodynamic situation of the patient (IIb,B).^19^

### Main studies

One of the first RCTs to approach the theme was conducted in an Italian center by Politi et al.. Two hundred and fourteen consecutive patients were excluded with multivascular AMI and CAD in the context of PPCI. Multivascular disease was defined as the presence of stenosis greater than 70% in two or more epicardial coronaries or their main branches, in visual angiographic evaluation. Before their first angioplasty, patients were randomized into three main strategies: PCI solely of the responsible artery, complete staged revascularization or complete revascularization at the moment of PPCI.^20^

The primary outcome considered consisted of greater cardiac events, defined by: cardiac and non-cardiac death, in-hospital mortality, reinfarction, rehospitalization due to acute coronary syndrome and need for a new revascularization. After follow-up of 2.5 years, at least one greater cardiac event was identified in 42 (50%) of the patients treated only with PCI of the responsible artery, in 13 (20%) of the group of complete staged revascularization and in 15 (23.1%) of the ones submitted to complete revascularization during PPCI, displaying significant difference (p < 0.001). In-hospital mortality, need for new revascularizations and rehospitalization occurred more frequently among patients treated with PCI solely of the responsible artery. There was no difference in relation to mortality among the three groups.

The PRAMI Study - Randomized Trial of Preventive Angioplasty in Myocardial Infarction,^17^ published in 2013, was the first RCT of greater magnitude to compare angioplasty of the artery responsible for the AMI to the complete revascularization of lesions in other arteries with no responsibility during the index procedure. This open-labeled study randomized 465 patients submitted to PPCI. Patients selected for complete revascularization, all angiographically stenosis greater than 50% were treated in the same procedure of the PPCI. The primary outcome considered consisted of deaths by all causes, non-fatal AMI or refractory angina. After an average follow-up of 23 months, the study was interrupted early due to a significantly high difference between the groups. There was reduction of 65% in the primary outcome in the group submitted to complete revascularization during PPCI (HR 0.35; 95%CI 0.21-0.58; p < 0.001). A similar reduction was also found when analyzing the primary outcome consisted of cardiac death or non-fatal AMI (HR 0.36; 95%CI 0.18-0.73). The group submitted to complete revascularization presented higher time of procedure and contrast volume, however complication rates, including CVA, bleeding and contrast-induced nephropathy were similar between the groups.

This was a study on preventive angioplasty, treating other lesions above 50%, in addition to the responsible coronary without worrying whether they were causing limitations to the flow. Critical reviews on this study point out its unblinded design as a flaw, once that patients were aware they had untreated lesions, thus making the findings susceptible to bias. Besides, patient in the control group were not tested for the presence of ischemia due to residual lesions, being investigated and treated only if refractory angina was observed. When compared to other studies on the same matter, it should be emphasized that the complete staged revascularization was not evaluated in a moment other than in PPCI.

Posteriorly, the CvLPRIT study - Complete versus Lesion-only Primary PCI Trial,^21^ RCT, open, multicentered, conducted in seven centers in the United Kingdom, was published in 2015. Two hundred and ninety-six patients with STEMI were randomized for PCI of the compromised artery or complete revascularization in index admission. Allocation into the groups was performed after angiography with stenosis greater than 50% in arteries not responsible for the AMI. The complete revascularization should be performed in the same procedure, though the operator could choose to take on the procedure at some other moment before hospital discharge, including, thus, the staged revascularization strategy.

After an average follow-up of 12 months, there was a significant reduction (55%) in the primary outcome consisted of mortality, recurrent AMI, HF or revascularization guided by ischemia (10% *versus* 21%; HR 0.45; p = 0.009) in patients submitted to complete revascularization. The reduction of the primary outcome was evident early within the first 30 days, although not significantly (p = 0.055).

Of the 150 patients selected for complete revascularization, 64% of them were treated during the same procedure as PPCI, subgroup in which a tendency to greater benefit was observed. It should be noted, however, that the study did not have a specific design for this kind of analysis.

As expected, patients submitted to complete revascularization had a greater number of stents implanted, as well as higher time of procedure and contrast volume used. The safety outcomes considered, including CVA, larger bleeding and contrast-induced nephropathy, were similar between the groups.

It should be noted that, although positive results have been found, the CvLPRIT Trial did not have sufficient statistical power to detect difference in important components of the primary outcome, such as death and AMI.

Furthermore, in attempting to get an answer on the best management of multivascular CAD in PPCI, Engstrom et al. carried out the DANAMI3-PRIMULTI study,^22^ published in 2015. This open RCT compared a complete revascularization strategy guided by a fractional flow reserve (FFR) two days after the index procedure with no additional intervention after PPCI. Six hundred and twenty-seven patients were randomized in two centers in Danmark, being considered as primary outcome a set of mortality by all causes, reinfarction or revascularization guided by ischemia (either subjective or objective) of arteries not related to the AMI.

After follow-up of 27 months, there was a significant decrease of 44% in the primary outcome analyzed (HR 0.56; 95%CI 0.38-0.83; p = 0.004) in the group submitted to PCI of the arteries not responsible for the AMI. However, when analyzed individually, mortality rates due to all causes and reinfarction were similar in both groups, while the complete revascularization group had better results because of the need for less reinterventions due to refractory angina.

The real benefits of complete revascularization are questioned here, once there were no differences in isolated harsh outcomes, such as mortality and reinfarction, and the fact that primary outcome reduction was due, primarily, to the need for new revascularizations.

In a metanalysis consisted of three RCTs carried out until September 2013, with a total of 748 patients (416 randomized for complete revascularization and 322 for PCI of the responsible artery), Pandit et al. showed the benefits of preventive PCI. In the group treated with complete revascularization during PPCI, there was a significant reduction in cardiovascular death (HR 0.39; 95%CI 0.18-0.83; p = 0.01), in the need for new revascularizations (OR 0.28; 95%CI 0.18-0.44; p = 0.00001) and in non-fatal AMI (OR 0.38; 95%CI 0.20-0.75; p = 0.005).^23^

Spencer et al. Carried out another metanalysis, in which all RCTs were included, until 2015, comparing complete revascularization *versus* PCI solely of the artery responsible for the AMI. Five clinical trials were included, with a total of 1,568 patients. Complete revascularization was associated to the reduced need for new revascularization (RR 0.36; 95%CI 0.27-0.49) and decreased recurrent AMI (RR 0.41; 95%CI 0.30-0.57). However, when considering the total mortality, there was no significant difference between the groups (RR 0.82; 95%CI 0.53-1.26).^24^

Tarantini et al. performed a systematic paired review and a network metanalysis with the objective of verifying which is the best therapeutic strategy in patients with ST-Elevation AMI and multivascular CAD. We included prospective and retrospective studies published between 2001 and 2015. Analyses were carried out for three intervention strategies: 1) PCI solely of the affected artery; 2) complete revascularization during PPCI procedure; and 3) complete staged revascularization during hospitalization.^25^ Thirty-two studies were included (13 prospective and 19 retrospective ones), with a total of 54,148 patients. The paired analysis showed the complete staged revascularization was associated to lower mortality in the short and long terms, when compared to PCI solely of the artery responsible and complete revascularization during PPCI. On the other hand, the revascularization solely of the responsible artery in relation to a complete revascularization during PPCI presented reduced mortality. In the network analysis, the complete staged revascularization associated consistently to improved survival rates. Table 1 describes the main findings of the RCTs presented.

**Table 1:** Main characteristics of randomized clinical trials compared to percutaneous coronary intervention solely of the responsible artery versus complete revascularization of acute myocardial infarction with ST Elevation

Study	Type of study	N	PCI non-responsible arteries	Primary outcome (composed)	Result (primary outcome)
Politi et al.	RCT	214	Angiography > 70%	Cardiac and non-cardiac death, in-hospital death, reinfarction, rehospitalization due to ACS, new revascularization	Reduction of greater events with complete revascularization (p < 0.001)
PRAMI	RCT	465	Angiography > 50%	Death by all causes, Non-fatal AMI, refractory angina	Reduction of 65% with complete revascularization (HR 0.35; 95%CI 0.21-0.58; p < 0.001)
CvLPRIT	RCT	296	Angiography > 50%	Mortality, recurrent AMI, CI, revascularization guided by ischemia	Reduction of 55% with complete revascularization (10% *vs* 21%; HR 0.45; p = 0.009)
DANAMI-3-PRIMULTI	RCT	627	FFR < 0.80	Mortality by all causes, reinfarction, revascularization guided by ischemia	Reduction of 44% with complete revascularization (HR 0.56; 95%CI 0.38-0.83; p = 0.004).
COMPARE-ACUTE	RCT	885	FFR ≤ 0.80	Mortality by all causes, non-fatal AMI, need fo any revascularization, cerebrovascular events	Reduction of 65% with complete revascularization (HR 0.35; 95%CI 0.22-0.55; p < 0.001)

RCT: randomized clinical trials; HR: hazard ratio; CI: confidence interval; ACS: acute coronary syndrome.

Recently, Smits et al. published the results of the Compare-Acute multicenter clinical trial, which randomized 885 patients with AMI and multivascular CAD submitted to PPCI. These patients were compared, in a ratio of 1:2, for the complete revascularization guided by FFR or PCI solely of the artery responsible for the AMI. We included patients with indication for PPCI and who had had other stenosis of, at least, 50% in quantitative or visual angiographic evaluation. All patients were submitted to FFR, however the ones selected for treatment of the affected artery and their assistant doctors were not informed on the results. In the randomized group, PCI of the arteries not responsible for the AMI would be conducted if ≤ 0.80, preferably during the same PPCI procedure. The operating doctor would be able to choose to perform a complete revascularization at another moment, provided it would be carried out during the hospitalization and within 72 hours.^26^

The primary outcome considered was the one consisted of death by all causes, non-fatal AMI, need for any revascularization and cerebrovascular events within 12 months. After one year, the primary outcome occurred in 23 patients of the complete revascularization group and in 121 of those treated only with PCI for the affected artery (HR 0.35; 95%CI 0.22-0.55; p < 0.001). There was no significant difference between the groups, when analyzed the isolated outcomes of death by all the causes and AMI. There was a significant decrease in the need for new revascularization in the complete revascularization group (HR 0.32; 95%CI 0.20- 0.54; p < 0.001). No differences were observed in relation to the safety outcomes analyzed.

Although with higher number of selected patients and also with the use of FFR functional evaluation, the study once again shows positive results at the cost of reducing the need for new revascularizations. Harsh outcomes, such as death and AMI, when analyzed in isolation, did not differ between groups.

## Discussion

Considering the results of the studies presented, the complete revascularization of arteries not responsible for the AMI seem to offer better results than just the clinical treatment. However, it should be noted they are studies with a small number of patients and with heterogeneous designs, having been used different criteria for the revascularization of non-responsible stenosis, whether angiographic or based on functional evaluation, such as the FFR. The small number of events observed in the studies available makes it rather difficult to be defined as for the benefits of this strategy in the mortality rates and recurrent infarction.

Initially published metanalyses and registries failed to demonstrate the clear benefit of complete revascularization on PCI solely of the responsible artery. In the study by Cavender et al.,^8^ this fact is possibly related to the greater severity of the patients treated with complete revascularization, most of them with reduced left ventricular ejection or in cardiogenic shock. Another limitation of these initial analyses is the great heterogeneity of the studies included, in addition to the absence of specific designs for the evaluation of greater cardiovascular outcomes, as in the study by Sethi et al.^10^ On the other hand, more recent metanalyses with greater number of studies, although still mostly observational, start to point in favor of the benefit of complete revascularization, especially when carried out in a staged way. This benefit is confirmed with the publication of the main RCTs which, despite their limitations, hade an appropriate design to evaluate the best strategy.

Another point to be considered is the appropriate moment to perform the complete revascularization - during the index procedure or in a staged way, during hospitalization. In the CvLPRIT study, patients treated in the index procedure had better results, despite the study not having been design for this evaluation. In DANAMI-3-PRIMULTI, patients were submitted to revascularization two days after the acute event, with reduced outcomes, although due to the need for new revascularizations. Logistic aspects of performing these multi-arterial procedures in patients with STEMI should be especially considered in our field, once they presuppose trained personnel, available material and clinical and surgical backup, which may not be available during procedures performed during late hours, for example.

At present, some RCTs are underway in an attempt to elucidate the best therapeutic strategy in this context. Among them, the COMPLETE study (Complete versus Culprit-only Revascularization to Treat Multi-vessel Disease After Primary PCI for STEMI trial),^27^ designed to detect differences in cardiovascular death or AMI with complete staged revascularization *versus* PCI solely of the responsible artery. There is also the FRAME-STEMI trial, which will evaluate the revascularization strategies guided by FFR of the lesion compared to the traditional angiographic evaluation.^28^

Despite the findings of the last clinical trials, there is still no definitive answer regarding the best treatment for multivascular CAD in STEMI and PPCI contexts.^29^ Many particularities should be considered in the decision on which is the best moment for the approach of non-responsible arteries, among which, clinical characteristics of the patient, as well as angiographic characteristics of the lesion and of non-related arteries. For instance, it could be more prudent to postpone the complete revascularization when the angioplasty of the lesion was complex, requiring great volumes of contrast or resulting in unsatisfactory final flow. All the same, complex lesions, such as bifurcations, chronic, highly calcified occlusions may be treated at a later time.^30^

The advantages and disadvantages of each intervention strategy should also be considered. The complete revascularization could be beneficial, once it allows for the fast reestablishment of blood flow, increasing the viable myocardial area and leading to an improvement of the left ventricular ejection fraction. In addition, it is related to reducing vascular complications through the lesser need for punctures and the reduced hospitalization time, promoting an increase in cost-effectiveness.

On the other hand, the disadvantages of a complete revascularization include prolonged procedure time, with greater exposure to radiation and greater use of contrast, potentiating the risk of nephropathy by contrast. There is also higher risk of acute or subacute stent thrombosis due to the prothrombotic and pro-inflammatory AMI scenario.^7^

## Conclusion

The multiarterial approach in a same procedure may be used in carefully selected patients, provided the situation described in the text presented are respected. An approach of the multiarterial treatment of all severe lesions in a step-by-step manner during the index hospitalization seems to be based on RCTs and in a great metanalysis recently published, and would be the alternative to be recommended in most cases considering the current evidence. However, until there is no definitive recommendation, the appropriate clinical judgement, of the interventionist along with the clinical cardiologist, remains as the standard strategy to be followed.
